# The *Pseudomonas aeruginosa* Catabolite Repression Control Protein Crc Is Devoid of RNA Binding Activity

**DOI:** 10.1371/journal.pone.0064609

**Published:** 2013-05-23

**Authors:** Tetyana Milojevic, Irina Grishkovskaya, Elisabeth Sonnleitner, Kristina Djinovic-Carugo, Udo Bläsi

**Affiliations:** 1 Department of Microbiology, Immunobiology and Genetics, Max F. Perutz Laboratories, University of Vienna, Vienna, Austria; 2 Department of Structural and Computational Biology, Max F. Perutz Laboratories, University of Vienna, Vienna, Austria; 3 Department of Biochemistry, Faculty of Chemistry and Chemical Technology, University of Ljubljana, Ljubljana, Slovenia; Florida International University, United States of America

## Abstract

The Crc protein has been shown to mediate catabolite repression control in *Pseudomonas*, leading to a preferential assimilation of carbon sources. It has been suggested that Crc acts as a translational repressor of mRNAs, encoding functions involved in uptake and breakdown of different carbon sources. Moreover, the regulatory RNA CrcZ, the level of which is increased in the presence of less preferred carbon sources, was suggested to bind to and sequester Crc, resulting in a relief of catabolite repression. Here, we determined the crystal structure of *Pseudomonas aeruginosa* Crc, a member of apurinic/apyrimidinic (AP) endonuclease family, at 1.8 Å. Although Crc displays high sequence similarity with its orthologs, there are amino acid alterations in the area corresponding to the active site in AP proteins. Unlike typical AP endonuclease family proteins, Crc has a reduced overall positive charge and the conserved positively charged amino-acid residues of the DNA-binding surface of AP proteins are partially substituted by negatively charged, polar and hydrophobic residues. Crc protein purified to homogeneity from *P. aeruginosa* did neither display DNase activity, nor did it bind to previously identified RNA substrates. Rather, the RNA chaperone Hfq was identified as a contaminant in His-tagged Crc preparations purified by one step Ni-affinity chromatography from *Escherichia coli*, and was shown to account for the RNA binding activity observed with the His-Crc preparations. Taken together, these data challenge a role of Crc as a direct translational repressor in carbon catabolite repression in *P. aeruginosa*.

## Introduction

Bacteria can utilize various compounds as a carbon and energy source, which facilitates growth and survival in different habitats as well as the adaptation to changing environmental conditions. The utilization of preferred carbon sources is mediated by a global regulatory mechanism termed catabolite repression (CR). CR ensures the preferential assimilation of one compound that supports efficient growth, inhibiting at the same time the uptake and/or expression of genes required for the metabolism of non-preferred compounds [1], [2]. The molecular mechanism of catabolite repression control has been extensively studied in enteric bacteria, where glucose is the preferred carbon source. In these organisms, enzymes of the phosphoenolpyruvate-dependent phosphotransferase system mediate catabolite repression control by regulation of the cyclic AMP (cAMP) level [3]. Unlike in *Escherichia coli*, in *Pseudomonas* species the adenylate cyclase activity and cAMP pools do not fluctuate with a given carbon source, nor does the addition of cAMP relieve repression of catabolite responsive pathways [4], [5]. In addition, only one sugar phosphotransferase system (fructose) has been identified in *Pseudomonas* [6].

The Crc (catabolite repression control) protein has been identified as a regulator of CR in *Pseudomonas* [2], [7]. *Pseudomonas crc* mutants failed to repress multiple degradative pathways when grown in the presence of tricarboxylic acid cycle intermediates [8]. Crc controls the sequential assimilation of amino acids and inhibits the expression of several genes involved in uptake and catabolism of sugars, alkanes and benzoate [2], [7], [9]. Crc was further shown to modulate biofilm formation [10], susceptibility to several antibiotics [11] and biosynthesis of the virulence factor pyocyanin [12]. Moreover, a *P. aeruginosa crc* mutant was impaired in swimming and swarming [11]. Thus Crc protein seems to operate at the crossroad of metabolism and virulence in *P. aeruginosa*.

It has been proposed that Crc exerts catabolite repression control by binding to A-rich motifs at or in the vicinity of the ribosome binding site of target mRNAs, thereby repressing their translation [13], [14], [15]. Translational repression by Crc was suggested to be alleviated by the regulatory RNAs CrcZ and CrcZ/CrcY in *P. aeruginosa* and *P. putida*, respectively [15], [16]. The CrcZ RNA was shown to be synthesized under conditions when the C:N ratio decreases [15]. These RNAs possess A-rich stretches and are believed to act by sequestering Crc, which in turn was suggested to result in expression of mRNAs repressed by Crc [15], [16]. However, it has also been hypothesized that Crc participates in signaling pathways controlled by phosphorylation/dephosphorylation events [17].

Crc shares sequence similarity (25 to 37% identity) with DNA repair enzymes belonging to the family of apurinic/apyrimidinic (AP) endonucleases of both Prokaryotes and Eukaryotes. However, Crc does not appear to have nuclease activity [18]. Here, we report the crystal structure of the *P. aeruginosa* Crc refined to 1.8 Å. At variance with a recent report [19] our structural analyses did not corroborate intrinsic nucleic acid binding properties of Crc. Using different DNA substrates, we show that Crc is devoid of DNA nuclease activity, which can be reconciled with an alteration of the catalytically active site when compared to the AP endonuclease family. In addition, by employing electrophoretic mobility shift assays (EMSA) we show that *P. aeruginosa* Crc protein purified to homogeneity does not bind to the previously identified RNA substrates *amiÈ* and *CrcZ`* [15], [16]. We provide evidence that the previously reported RNA-binding properties of Crc are attributable to contaminations of the Ni-affinity purified Crc preparations with the *E. coli* RNA chaperone Hfq. Hence, although there is convincing evidence for an involvement of *P. aeruginosa* Crc in CR, *per se* it does not appear to exert this function by binding to and acting as a translational repressor on target mRNAs.

## Results and Discussion

### The structure of *P. aeruginosa* Crc provides a clue for the absence of nuclease activity

**Figure 1:**
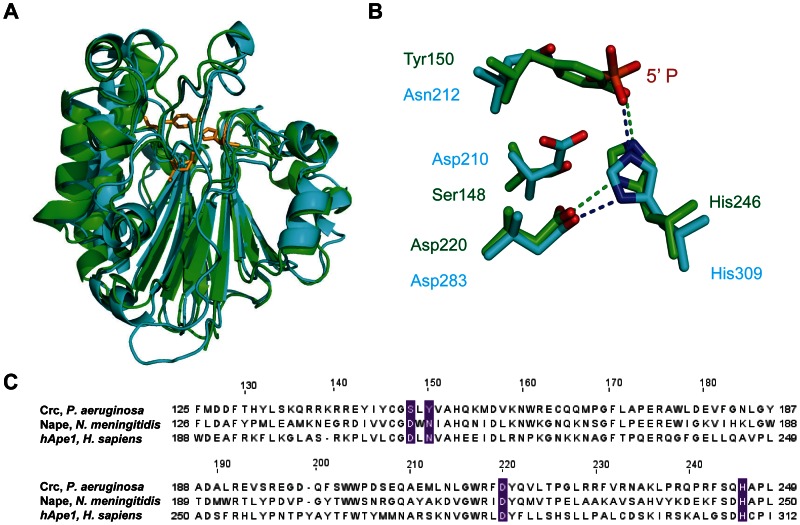
Structural comparison of Crc with AP endonucleases. (**A**), Superposition of the ribbon diagrams of Crc and its ortholog hApe1. Crc and hApe1 are colored in green and cyan, respectively. The positions of amino acid residues corresponding to active site residues in AP proteins are depicted in orange. (**B**), The catalytically active site of hApe1 (PDB accession code 1DE8) (cyan) is superposed with the corresponding area of Crc (green). (**C**), Sequence alignment of Crc with hApe1 (*Homo sapiens*) and Nape (*Neisseria meningitidis*). The four highly conserved residues located at the catalytically active site are highlighted in pink.

The crystals of Crc diffracted to 1.8 Å resolution and belonged to space group *P3_2_21* (unit-cell parameters a = b = 74.66, c = 123.16 Å), with the asymmetric unit containing one molecule. The structure of Crc was refined to a crystallographic R_work_ and R_free_-values of 19.4 and 22.0%, respectively (Table 1). Crc is a compact, globular αβ-protein, consisting of two six-stranded β-pleated sheets flanked by six α-helices forming a four-layered αβ-sandwich motif (Figure 1A). The overall structure of Crc shares high similarity with members of the AP endonuclease protein family. Although the amino acid sequence identities between Crc and these proteins are only 25 to 37% (Figure S1), the superposition of Crc with the available structure of the AP endonuclease hApe1 (PDB accession code 1BIX, Figure 1A) revealed high structural similarity with a root mean square deviation (RMSD) of 1.59 Å over 253 superposed Cα atoms. The comparison of the Crc structure obtained in this study with a recently published structure of Crc (PDB accession code 4F1R) resolved at 2.20 Å resolution [19] revealed only minimal differences with a RMSD of 0.26 Å over 259 superposed Cα atoms.

**Table 1:** Data collection and refinement statistics.

DATA COLLECTION	
Source	ID231, ESRF
Wavelength (Å)	0.9
Resolution (Å)	44.59–1.80 (1.84–1.80)^a^
Space group	P3_2_21
Unit cell (Å, °)	a = b = 74.66, c = 123.16α = β = 90, γ = 120
Molecules/a.u.	1
Unique reflections	37261 (2073)
Completeness (%)	99.6 (96.9)
R_merge_ *^b^*	0.104 (0.833)
R_meas_ *^c^*	0.109 (0.910)
R_pim_ *^d^*	0.032 (0.355)
Multiplicity	11.0 (6.1)
I/sig(I)	13.9 (1.7)
B_Wilson_ (Å^2^)	31.6

REFINEMENT	
R_cryst_ *^e^*/R_free_ *^f^*	19.4/22.0
R.m.s.d. bonds (Å)	0.02
R.m.s.d. angles (°)	2.02

a Values in parentheses are for the highest resolution shell.

b
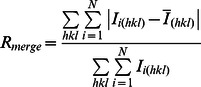

c
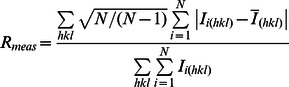

d
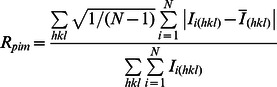

Where 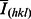 is the mean intensity of multiple 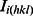 observations of the symmetry-related reflections, N is the redundancy.

e
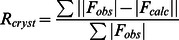

f R_free_ is the cross-validation R_factor_ computed for the test set of reflections (5%) which are omitted in the refinement process.

**Figure 2:**
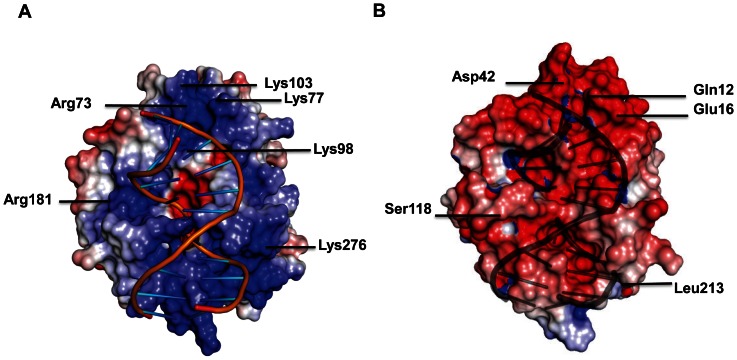
Electrostatic potential of the DNA binding surface of hApe1 and the corresponding surface area of Crc . The color coded electrostatic surface potential of hApe1 (**A**) and Crc (**B**) was drawn using the Adaptive Poisson-Boltzmann Solver package [37] within PYMOL [36]. The electrostatic potential ranges from −5 (red) to +5 (blue) kT/e. The path of the DNA is shown in orange for hApe1 (**A**) and superimposed on Crc (**B**).

Apurinic/apyrimidinyc endonucleases are central elements of the excision DNA repair mechanism. These enzymes excise abasic residues from the DNA backbone that arise from DNA damage by cleaving the DNA 5` to the abasic site, leaving a 3`-hydroxyl group [20]. Four conserved catalytically active residues (Asp210, Asn212, Asp283 and His309 for hApe1), located in the cleft on the edge of the αβ-sandwich, are a determinant for AP endonuclease activity. Among them only two (Asp220 and His246) are present in Crc (Figure 1B and C). A closer analysis of the Crc structure revealed crucial differences as compared with the previously reported structure of the human base excision repair enzyme (hApe1) in complex with DNA (PDB accession code 1DE8) [21]. The bulky side-chain of Tyr150 (corresponds to Asn212 in hApe1) protrudes into the active site, occupying the position of the scissile phosphate group of the DNA backbone in the apurinic/apyrimidinic site (Figure 1B). Moreover, in the active site of hApe1 Asp210 is favorably positioned with the phosphate group to serve as a general acid for hydrolysis. The substitution of this residue to a serine in Crc makes DNA hydrolysis rather unlikely (Figure 1B), which is consistent with the previously reported lack of exo- and endonuclease activity of Crc [18]. In addition, the positive electrostatic potential along the DNA-binding area in hApe1 predominantly results from six highly conserved, positively-charged residues, Arg73, Lys77, Lys98, Lys103, Arg181 and Lys276 (Figure 2A and S1), which contribute to the electrostatic attraction of the DNA backbone. With the exception of Arg37 (corresponding to Lys98 in hApe1) these positively charged residues are not present in Crc. They are substituted by the negatively charged Glu16 and Asp42 and the non-charged Gln12, Ser118 and Leu213 residues (Figure 2B). Most likely, this results in a reduced capacity of Crc to attract negatively charged nucleic acids (Figure 2A and B).

His-Crc was purified to homogeneity (Figure S2A) by one-step Ni-affinity chromatography (NAC) followed by size exclusion chromatography (SEC) from *P. aeruginosa* strain PAO1 (pME9670). Three double stranded DNA substrates were then used to revisit the nuclease activity of Crc: (i) 25U consists of a random sequence with an U-A pair at position 11, which has been used to test for exonuclease activity of Nape from *N. meningitides* [20]; (ii) 25AP was used to study a possible endonuclease activity. It was generated by treating 25U with uracil DNA glycosylase (UDG) to create an abasic site at the position previously occupied by uracil [20]; (iii) phzM_DNA_ containing a 20 base-pair fragment of *phzM* comprising base-pairs from position -10 to +10 with regard to the A (+1) of the start codon. *E. coli* exonuclease III was used as a positive control. Crc was not able to cleave/degrade any of these double stranded DNA fragments (Figure S2B-S2D). Even after increasing the incubation time to 120 min and the concentration of Crc to 20 µM, which represented a 1000-fold excess over the nucleic acid substrates, no nuclease activity was observed (not shown). In addition, we tested whether Crc can cleave/degrade (i) a RNA-DNA hybrid comprising a stretch of *phzM* mRNA (−10 to +10 with regard to the A (+1) of the start codon) and the complementary DNA strand, as well as (ii) the same segment of *phzM* mRNA only. In both cases no cleavage/degradation of the substrates was observed (Figure S2E-S2F). Taken together, although the overall structure of Crc has a high homology to AP nucleases, the function of Crc is obviously distinct from this family, which is also corroborated by the structural data with respect to Ser148 and Tyr150. It could be argued that specific cofactors required for Crc function are missing in the *in vitro* assay. However, at least in the presence the following molecules AMP, ADP, ATP, cAMP, GTP, NAD-NADH, NADP-NADPH, glutathione and acetyl coenzyme A no nuclease activity was observed [18].

### Crc protein purified to homogeneity displays no RNA binding activity

**Figure 3:**
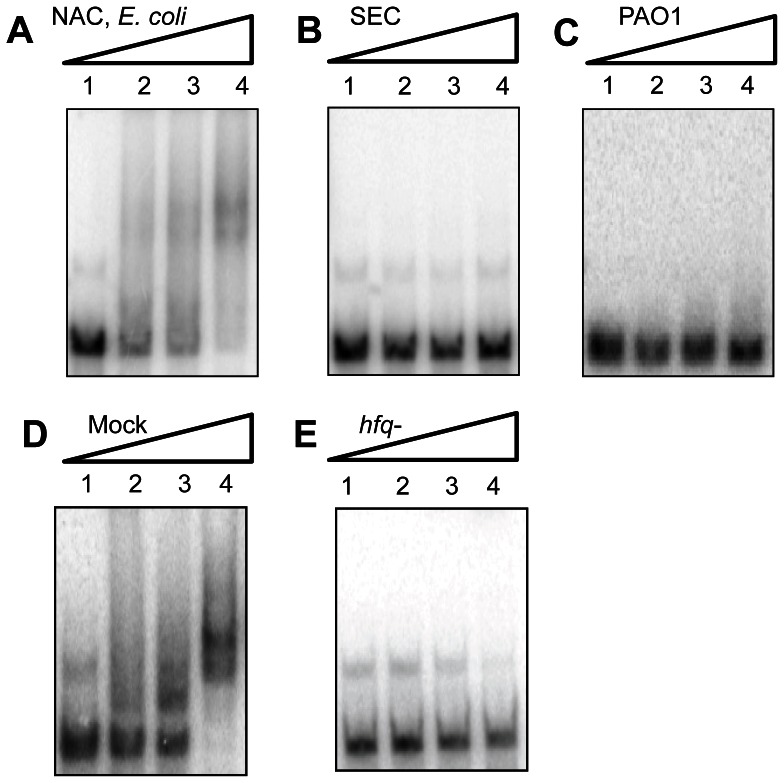
Electrophoretic mobility shift assays using CrcŹ RNA. Electrophoretic mobility shift assay of 10 nM 5′-end labeled CrcZ′ RNA with increasing amounts of His-Crc purified from the *E. coli* strain Rosetta™ (DE3)(pLysS pETM14lic-His_6_Crc) by one-step NAC (**A**) and by one-step NAC followed by SEC (**B**), respectively. EMSA assay employing the His-Crc protein purified from the *P. aeruginosa* strain PAO1(pME9670) by one-step NAC (**C**), the protein eluate obtained after one-step NAC from strain Rosetta™ (DE3)(pLysS, pETM14lic) (mock; no Crc protein) (**D**) and the His-Crc protein from the *E. coli hfq-* strain JW4130(pME9670) by one-step NAC (**E**). Lane 1, no protein was added to labeled *CrcZ`* RNA. Lanes 2-4, the protein fractions were added in 50, 100 and 200-fold molar excess over labeled RNA. In the case of the mock preparation (**D**), the same amount of protein was added to RNA as in the experiments shown in panels **A**, **B** and **C**.

Several studies have put forward the hypothesis that Crc binds to RNA, in particular to A-rich motifs in the translation initiation region of several *P. aeruginosa* [12], [15] and *P. putida* [13], [14] mRNAs as well as to the regulatory RNAs CrcY and CrcZ [15], [16] that have been suggested to antagonize the function of Crc in post-transcriptional control [15]. However, the observed Kd values for these substrates were rather low [12]. In all experiments, His-tagged Crc preparations of both *P. aeruginosa* and *P. putida* obtained from *E. coli* were used. During the course of our studies we attempted to obtain co-crystals between Crc protein and a part of the 5′untranslated region of *P. aeruginosa amiE* mRNA, encoding an aliphatic amidase. However, using Crc protein purified to homogeneity we were unable to reproduce the previously observed binding of Crc to *amiE* mRNA as well as to the regulatory RNA CrcZ. We therefore revisited the proposed RNA binding activity of Crc. As observed before [15], His-Crc protein purified by one-step Ni-affinity chromatography (NAC) did appear to bind to CrcZ` RNA, causing a band-shift (Figure 3A). However, when His-Crc was further purified by means of size exclusion chromatography (SEC), RNA binding was no longer observed (Figure 3B). Interestingly, His-tagged Crc purified from *P. aeruginosa* PAO1 by one-step NAC did likewise not bind to CrcZ` RNA (Figure 3C). Similar results were obtained when instead of CrcZ` RNA a stretch of *amiE* RNA, *amiÉ*, was used (Figure S3A –S3C) to which His-Crc protein purified by one-step NAC from *E. coli* was previously shown to bind [15].

### Hfq impurities cause false positive RNA-binding of His-Crc

**Figure 4:**
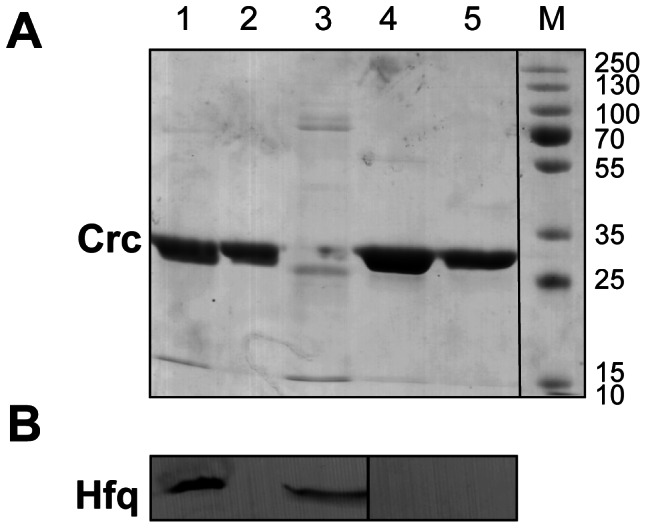
Purity of different His-Crc preparations and contamination with Hfq. (**A**) 12% SDS-polyacrylamide gel stained with Coomassie brilliant blue after electrophoretic separation of marker proteins (M; the numbers denote molecular masses in kD), His-Crc purified by one-step NAC (lane 1), His-Crc purified by NAC followed by SEC (lane 2), NAC eluate obtained from the mock control (no Crc protein) (lane 3), His-Crc purified by one-step NAC from the *hfq-* strain (lane 4) and His-Crc purified by one-step NAC from PAO1 (lane 5). (**B**) Immunodetetction of Hfq. The samples shown in A, lanes 1–3, were 5-fold concentrated and subjected to western-blotting using Hfq-specific antibodies.

The *E. coli* RNA chaperone Hfq is a hexameric protein that belongs to the eukaryal and archaeal family of Sm- and Sm-like proteins [22]. At least in enteric bacteria many small regulatory RNAs associate with Hfq and often require the protein for regulation of target mRNAs [22]. The Hfq proteins of different bacteria posess an evolutionarily conserved core consisting of amino-acid residues 7–66, whereas there is considerable variation at the C-terminus. The *E. coli* Hfq is characterized by a long and structurally disordered C-terminus [23], which harbors 4 histidine residues per subunit. In the hexameric protein this totals to 24 histidines, which allows metal-affinity purification without recombinant addition of histidines [23]. Thus, *E. coli* Hfq is one of the frequent contaminant proteins, which bind to Ni-NTA affinity columns. As the *amiÉ* and CrcŹ RNAs contains A-rich stretches [15] that can potentially serve as high affinity Hfq binding sites [24], we analyzed the His-Crc preparation obtained after one-step NAC as well as the samples obtained at different stages of the His-Crc preparation for the presence of Hfq using mass spectrometry. These analyses revealed multiple contaminations of the His-Crc preparation after NAC, which were removed during subsequent gel filtration (not shown). Among these impurities Hfq was detected. Hfq was also detected by western-blot analysis in the His-Crc preparation obtained from *E. coli* strain Rosetta™ (DE3) (pLysS, pETM14lic-Hfq_6_Crc) after one step NAC (Figure 4B, lane 1) but was absent in the His-Crc preparation obtained after SEC (Figure 4B, lane 2). To verify that the RNA-binding activity in the His-Crc preparation obtained from *E. coli* after NAC was caused by Hfq, one-step NAC was performed with extracts of the Hfq proficient *E. coli* strain Rosetta™ (DE3)pLysS transformed with plasmid pETM14lic (mock control; no Crc protein) as well as with extracts obtained from the *E. coli hfq-* strain JW4130 transformed with plasmid pME9670, encoding His-Crc [15]. As shown in Figure 3D, the protein fraction (see Figure 4, lane 3) obtained after NAC from the mock control strain (Hfq+/Crc−) was proficient in *CrcZ`* RNA binding, whereas the His-Crc fraction obtained after NAC from the *hfq-* strain was not (Figure 3E). In agreement, Hfq was detected by western-blot analysis in the protein fraction obtained from the mock-control strain (Figure 4B, lane 3). Again, analogous results were obtained when *amiÉ* RNA was used as a substrate in EMSA assays with the latter protein preparations (Figure S3D–S3E). Hence, these studies verified that the RNA binding activity present in the NAC fractions is due to Hfq and not to Crc.

Obviously, a problem encountered with the His-tag technology is the co-purification of endogenous proteins containing adjacent histidine residues [25]. While these impurities may not affect the specific activity/function of certain proteins, they may interfere with the activity of proteins that interact with various ligands, e.g. RNA binding proteins. The His-Crc protein was highly enriched by NAC in the preparation shown in Figure 4A, lane 1, whereas Hfq was not visible after Coomassie staining and could be only detected after immunostaining (Figure 4B, lane 1). Nevertheless, the low Hfq impurities were apparently sufficient to cause a band-shift with both, *amiÈ* and CrcZ` RNA (Figure 3A and Figure S3A). As Hfq has Kd values in the lower nanomolar range for A-rich substrates [24], this can be explained by A-rich stretches in the 5′UTR of *amiÉ* RNA as well as in CrcZ`[15]. To avoid false positive results, the purification of RNA binding activities by NAC should therefore be either performed using *E. coli hfq-* strains or bacteria, which possess Hfq proteins which are devoid of C-terminal histidine residues. The C-terminus of the Hfq protein of *P. aeruginosa* is shorter and devoid of His-residues [26]. This can readily explain why Hfq was not co-purified by one-step NAC, when His-Crc was purified from *P. aeruginosa* (Figure 4, lane 5), and thus why this His-Crc preparation was devoid of RNA-binding activity (Figure 3C).

## Conclusions

As Crc is apparently devoid of RNA-binding activity, but is clearly involved in regulating CR and virulence functions [2] our study challenges its proposed role in CR as a translational repressor. This function appears to be also questionable in light of the results of a recent proteome study. Among 65 proteins identified as being regulated by Crc, the translation initiation region of 50 corresponding mRNAs did not display A-rich stretches [11]. In addition, our attempts to co-immunoprecipitate RNAs using Crc specific antibodies yielded insufficient amounts of RNA for RNAseq (E. Sonnleitner, unpublished). This poses the question as to the molecular mechanism underlying Crc function. *P. aeruginosa crc* is constitutively expressed [2] and the Crc levels appear not to vary with growth phase or with the carbon source used [15], [17]. As CR is rapidly established upon a switch to a preferred carbon source [27] the possibility exists that Crc is reversibly activated/deactivated. Crc shows homology (44-48% identity) to several bacterial putative histidine kinases (ref|YP_003071874.1|, ref|YP_529138.1|, ref|ZP_09503924.1|) with a histidine residue at position 246 conserved among Pseudomonas spp. According to the structural data His246 is located at the interface of two parts of the Crc αβ-sandwich within a solvent exposed surface and corresponds to the catalytically active residues of all active members of AP endonuclease family (Figure 1B and S4). Given the importance of histidine phosphorylation-dephosphorylation events in CR of other bacteria [1], we are currently studying whether His246 in Crc is subject to phosphorylation.

## Materials and Methods

### Cloning of *crc* and purification of the His-Crc protein

A DNA fragment containing the full-length coding region of Crc protein from *Pseudomonas aeruginisa* PAO1 was amplified by PCR using *P. aeruginisa* PAO1 genomic DNA as template and primers R81_crcfw (5′-CCA GGG GCC CGC CAT GCG GAT CAT CAG TGT GAA C-3′) and S81_crcrev (5′-GAC CCG ACG CGG TTA TCA GAT GCT CAA CTG CCA GTC-3′) to generate overhangs for Ligase Indepedent Cloning (LIC), using single stranded complementary sequences created by T4-DNA polymerase (Fermentas). The PCR product was inserted into a modified pETM14 vector (EMBL, Heidelberg, Germany) carrying an N-terminal hexa-histidine tag sequence followed by the GST-HRV14-3C “PreScission” protease cleavage site. The correct *crc* insertion was verified by DNA sequencing. In the resulting plasmid pETM14lic-His_6_Crc, the *crc* gene is under transcriptional control of a T7 promoter and inserted in a manner that the corresponding protein is fused to an N-terminal cleavable His_6_-tag. The synthesis of the His-Crc protein in *E. coli* strain Rosetta™ (DE3)(pLysS, pETM14lic-His_6_Crc), in the *E. coli hfq*- strain JW4130(pME9670) [15], [28] and in *P. aeruginosa* PAO1(pME9670) [15] grown at 37°C in LB medium supplemented with 0.4% glucose was induced by addition of 1 mM IPTG for 3 h at 37°C. The cells were then harvested and the cell pellet was resuspended in lysis buffer (50 mM NaH_2_PO_4_ pH 8.0, 300 mM NaCl, 1 mM PMSF and 10 mM imidazole), and then lysed by sonication. His-Crc was purified by Ni-affinity chromatography (NAC) following a standard protocol. The His-Crc protein fraction obtained by NAC was further purified by Superdex-75 size-exclusion chromatography using a buffer containing 50 mM NaH_2_PO_4_ pH 8.0, 150 mM NaCl.

For crystallization, the Crc protein was purified from *E. coli* strain Rosetta™ (DE3)(pLysS pETM14lic-His_6_Crc) by NAC using a standard protocol and buffer containing 50 mM Hepes pH 8.0, 300 mM NaCl, 1 mM PMSF, 10 mM imidazole. The His-tag was removed with GST-HRV14-3C “PreScission” protease, followed by separation of Crc and “PreScission” by an additional round of Ni- and GST-affinity chromatography and Superdex-75 size-exclusion chromatography in 50 mM Hepes pH 8.0, 150 mM NaCl. Aliquots of the purified protein were concentrated to 6 mg/ml, either used immediately or frozen in liquid nitrogen, and stored at −80°C for further use. The purity of the protein solution used in the crystallization experiments was tested by SDS–PAGE analysis.

### Crystallization, data collection, structure determination and refinement

Crystals of Crc were initially obtained in the PEGRx HT crystallization screen (Hampton Research, Aliso Viejo, CA), using the sitting-drop vapor diffusion technique and a nanodrop-dispensing robot (Phoenix RE; Rigaku Europe, Kent, United Kingdom), and optimized to 40 mM Bis-Tris, pH 6.5, 10% w/v PEG 1500 using the hanging drop vapor diffusion technique at 22°C. For cryoprotection, the crystals were transferred to a solution containing 30% glycerol, before being flash cooled in liquid nitrogen. The diffraction data set was collected at the ESRF Synchrotron (Grenoble) at beamline ID23-1 at 100 K using a wavelength of 0.9 Å and processed using the XDS package [29], converted to mtz format using POINTLESS and scaled with SCALA [30].

The structure was solved by molecular replacement using the program PHASER [31] using the atomic coordinates of apurinic apyrimidinic endonuclease (Nape) from *Neisseria meningitidis* (PDB accession code 2JC5) as a search model. The structure was refined using the program REFMAC [32] and Phenix Refine [33], and rebuilding was done using the program Coot [34]. Coordinates have been deposited in the protein data bank (PDB accession code 4JG3). Data collection and refinement statistics are reported in Table 1. Stereo-chemistry and structure quality were checked using the program MolProbity [35]. The figures were produced using the program Pymol [36] and electrostatic surface potential was drawn using the Adaptive Poisson-Boltzmann Solver package [37].

### Nuclease assays

HPLC purified oligonucleotides Z94_25U (5′-GGATCACTATUATAGGTAGTTTAT-3′) and X94_phzM (5′-AGAATAAAAGATGAATAATT-3′) as well as the synthesised RNA fragment phzM` (5′-AGAAUAAAAGAUGAAUAAUU-3′) (Sigma) were 5′-end labeled with [γ^32^P]-ATP (Hartmann Analytic) and T4 polynucleotide Kinase (Fermentas). Double stranded DNAs and the RNA-DNA hybrid were made by mixing the 5′-end labeled oligonucleotides with equal concentrations of the complementary strand heated to 80°C for 5 min, followed by 5 min at 50°C, 37°C, 25°C and finally cooled down on ice. The efficient formation of the double stranded DNA substrates 25U and phzM_DNA_ as well as the RNA-DNA hybrid phzM_RNA-DNA_ was verified by assaying the samples on a native 15% polyacrylamide (PAA) gel. To generate the abasic 25AP DNA, 5 pmol 25U was incubated with 5U uracil DNA glycosylase (UDG; NEB) at 37°C for 30 min. All DNA fragments were purified with the nucleotide removal kit (QIAGEN), whereas the RNA and RNA-DNA hybrids were purified by phenol chloroform extraction. The nuclease assays were performed in exonuclease III reaction buffer (66mM Tris pH 8.0, 0.66mM MgCl_2_; Fermentas) with one pmol substrate and 50 pmol Crc or 200U *E.coli* exonuclease III (Fermentas) at 25°C. Aliquots were removed at the times indicated in the legends to the figures, and the reaction was terminated in formamide loading buffer (0.01% xylene cyanol, 0.01% bromphenol blue, 30 mM EDTA in formamide) heated for 10 min at 70°C before separation by denaturing PAA gel electrophoresis. An equivalent of 50 fmol of labeled substrate per lane was loaded onto the 15% PAA gels. Radioactively labeled bands were visualized using a PhosphoImager.

### Electrophoretic mobility shift assay (EMSA)

The RNAs were transcribed *in vitro* using T7 RNA polymerase (Epicentre) and PCR fragments as templates. The *amiE* ´ RNA template, containing the first 154 nt of the *amiE* transcript and the *CrcZ* ´ RNA template, containing the first 151 nt of the *CrcZ* transcript, have been described [15]_._ The RNAs were dephosphorylated with FastAP*®* thermosensitive alkaline phosphatase (Fermentas), and subsequently 5′ end-labeled using [γ-^32^P]-ATP (Hartmann Analytic) and polynucleotide kinase (Fermentas). The labeled RNAs were diluted to a concentration of 0.05 pmol/μl and added to unlabeled RNA of the same concentration in a 1∶50 ratio. 2 µl of substrate RNA (10nM final concentration) were incubated in 10 µl with increasing amounts of purified Crc in 10 mM Tris-HCl pH 8.0, 10 mM sodium phosphate pH 8.0, 10 mM MgCl_2_, 60 mM NaCl, 10 mM dithiothreitol and 25 ng tRNA to reduce non-specific interactions. In the case of the mock preparation the same amount of protein was added, which resulted in degradation of the RNA when higher protein concentrations were used. Therefore 5U Ribo-Lock *®* RNase inhibitor (Fermentas) was included in each reaction. The reaction mixtures were incubated at 37°C for 30 min to allow protein–RNA complex formation. Immediately before loading, the samples were mixed with 25% glycerol to a final concentration of 5% and loaded on native 4% polyacrylamide gels. Electrophoresis was performed in TBE buffer at 15 mA. Radioactive labeled bands were visualized using a PhosphoImager.

### Analysis of His-Crc preparations by SDS-polyacrylamide electrophoresis and immunodetection of Hfq

The protein preparations were analyzed on 12% SDS polyacrylamide gels stained with 0.3% Coomassie brilliant blue. In addition, equal amounts of His-Crc obtained by one-step NAC, His-Crc obtained by NAC followed by SEC, eluate obtained from the mock control strain (Hfq+/Crc−), His-Crc obtained from the *hfq-* strain and purified Hfq protein were subjected to electrophoresis on 12% SDS polyacrylamide gels. The proteins were then transferred to nitrocellulose membranes (Schleicher and Schuell) by electroblotting. Hfq protein was detected by immonoblotting as described previously [38].

## Supporting Information

Figure S1 **Sequence alignment of Crc with Nape (** ***Neisseria meningitidis*** **) and hApe1 (** ***Homo sapiens*** **).** The four highly conserved residues located at the catalytically active site are marked in pink; conserved positively charged amino acids along the DNA-binding area are boxed.
(TIF)

Figure S2 **Nuclease assays using Crc protein purified from** ***P. aeruginosa*** **.** (**A**) Purity of *P. aeruginosa* Crc protein purified from PAO1(pME9670). 12% SDS-polyacrylamide gel stained with Coomassie brilliant blue of marker proteins (M; the numbers denote molecular masses in kD), Crc protein after one-step NAC (NAC) and Crc protein after one-step NAC followed by SEC (SEC). 1 pmol of the substrates 25U (**B**), 25AP (**C**), phzM_DNA_ (**D**), phzM_RNA-DNA_ (**E**) and phzM_RNA_ (**F**) were incubated with 200U ExoIII (lanes 1–5) or with 50 pmol Crc (lanes 6–10) in a 50 µl reaction volume. Aliquots were removed at different times (lane 1 and 6: 0 sec, lane 2 and 7: 10 sec, lane 3 and 8: 50 sec, lane 4 and 9: 10 min lanes 5 and 10: 50 min) and separated on a 15% denaturing PAA gel.
(TIF)

Figure S3 **Electrophoretic mobility shift assay using** ***amiÈ*** **RNA.** Electrophoretic mobility shift assay of 10 nM 5′-end labeled *amiE*′ RNA with increasing amounts of His-Crc purified from the *E. coli* strain Rosetta™ (DE3)(pLysS, pETM14lic-His_6_Crc) by one-step NAC (**A**) and by one-step NAC followed by SEC (**B**), respectively. EMSA assay employing the His-Crc protein purified from the *P. aeruginosa* strain PAO1(pME9670) by one-step NAC (**C**), the protein eluate obtained after one-step NAC from Rosetta™ (DE3)(pLysS, pETM14lic) (mock control; no Crc protein) (**D**) and the His-Crc protein from *E. coli hfq*- strain JW4130(pME9670) by one-step NAC (**E**). Lane 1, no protein was added to labeled *amiE* ` RNA. Lanes 2–4, the protein fractions were added in 50, 100 and 200-fold molar excess over labeled RNA. In the case of the mock preparation (**D**), the same amount of protein was added to RNA as in the experiments shown in panels **A**, **B** and **C**.
(TIF)

Figure S4 **Ribbon diagram and surface of Crc highlighting the position of His246.** The position of His246 is depicted in the ribbon diagram (**A**) and in the surface representation (**B**) of Crc, suggesting its side chain localization within a solvent exposed area.
(TIF)
