# Supplementary figures and images for: The Pseudomonas aeruginosa Catabolite Repression Control Protein Crc Is Devoid of RNA Binding Activity
Source: PLoS One. 2013 May 23;8(5):e64609. doi: 10.1371/journal.pone.0064609 (PMC3662782; doi:10.1371/journal.pone.0064609)

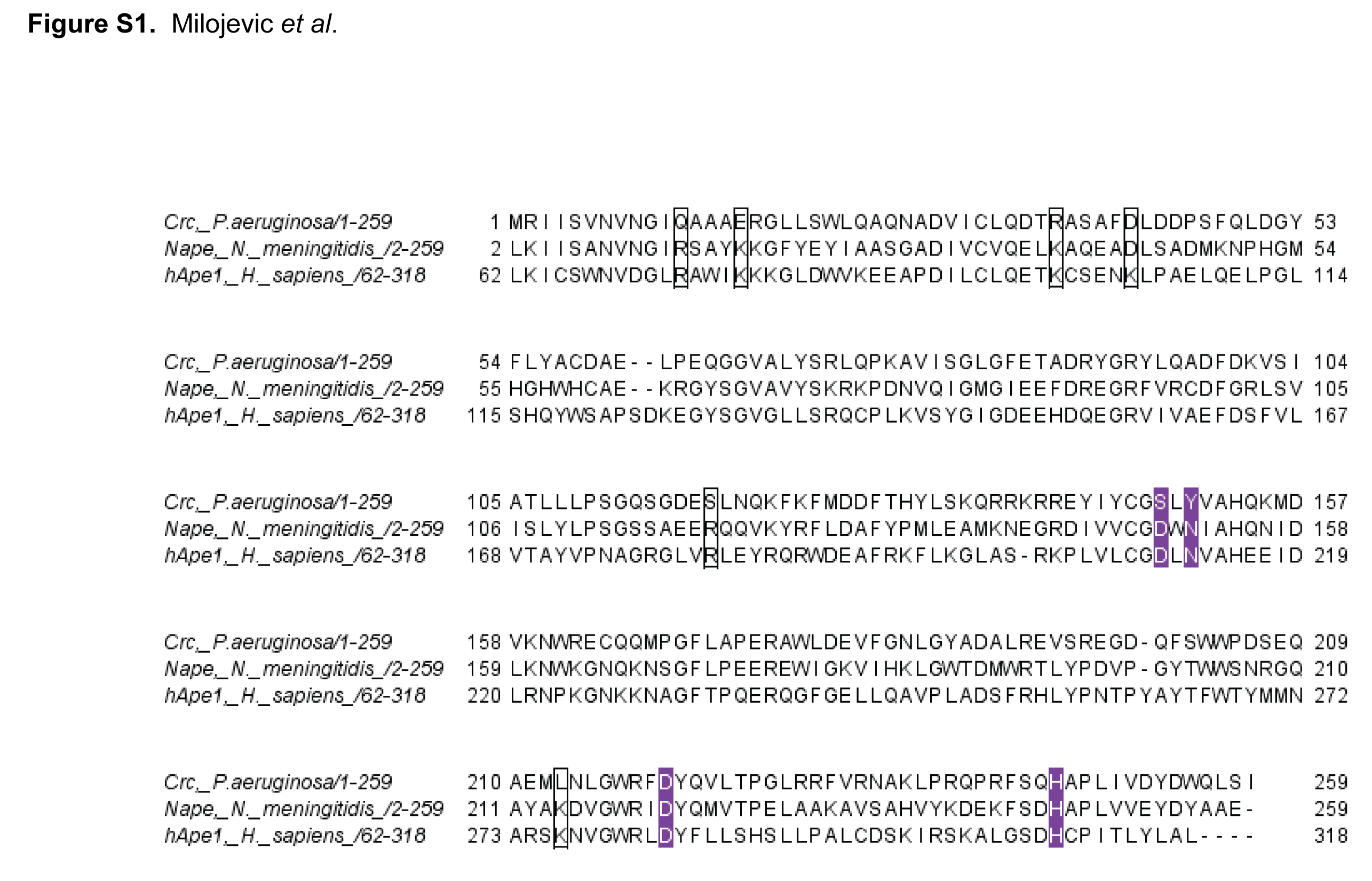

Supplement: Figure S1 — Sequence alignment of Crc with Nape ( Neisseria meningitidis ) and hApe1 ( Homo sapiens ). The four highly conserved residues located at the catalytically active site are marked in pink; conserved positively charged amino acids along the DNA-binding area are boxed. (TIF) [file pone.0064609.s001.tif]

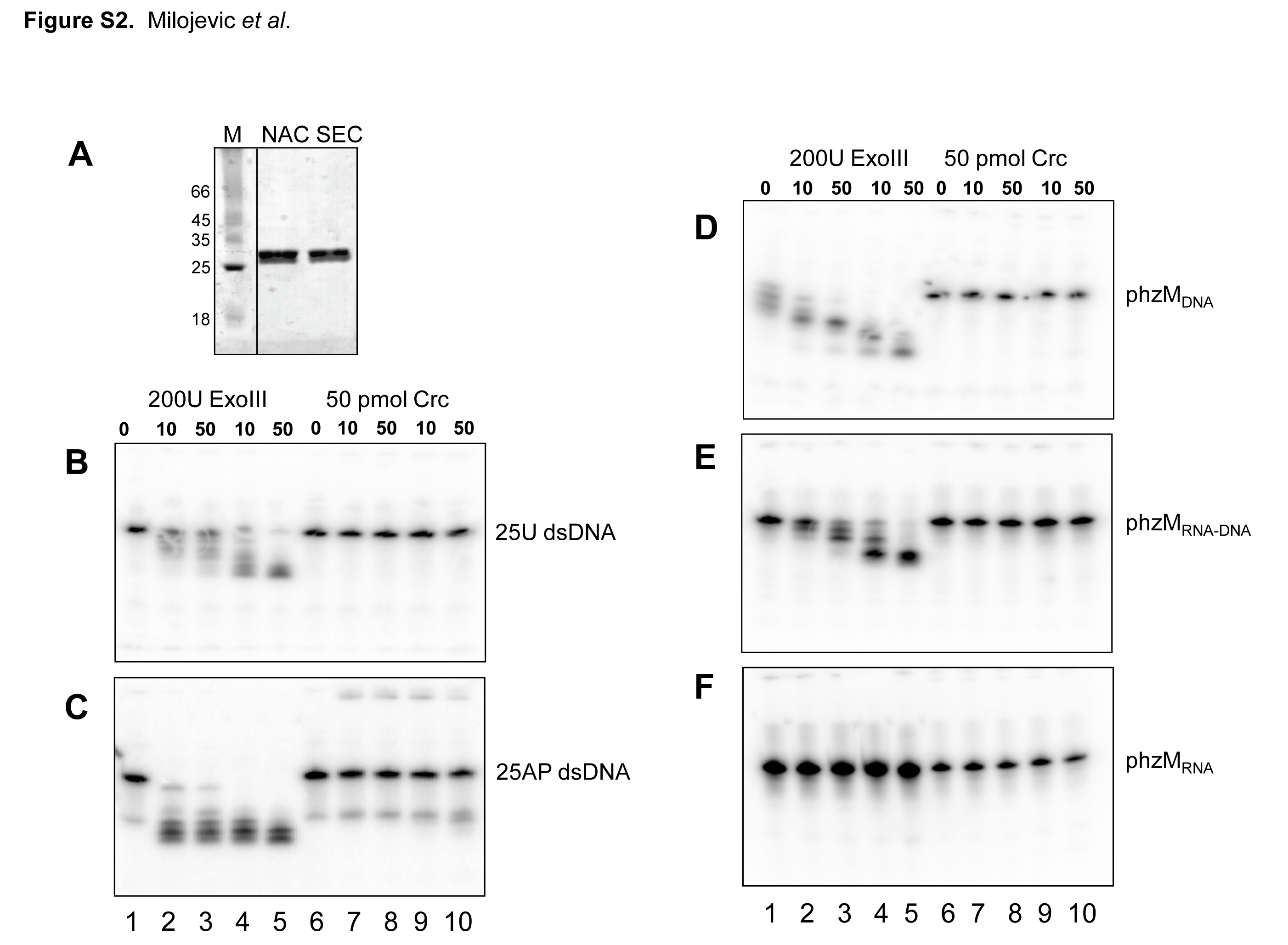

Supplement: Figure S2 — Nuclease assays using Crc protein purified from P. aeruginosa . (A) Purity of P. aeruginosa Crc protein purified from PAO1(pME9670). 12% SDS-polyacrylamide gel stained with Coomassie brilliant blue of marker proteins (M; the numbers denote molecular masses in kD), Crc protein after one-step NAC (NAC) and Crc protein after one-step NAC followed by SEC (SEC). 1 pmol of the substrates 25U (B), 25AP (C), phzMDNA (D), phzMRNA-DNA (E) and phzMRNA (F) were incubated with 200U ExoIII (lanes 1–5) or with 50 pmol Crc (lanes 6–10) in a 50 µl reaction volume. Aliquots were removed at different times (lane 1 and 6: 0 sec, lane 2 and 7: 10 sec, lane 3 and 8: 50 sec, lane 4 and 9: 10 min lanes 5 and 10: 50 min) and separated on a 15% denaturing PAA gel. (TIF) [file pone.0064609.s002.tif]

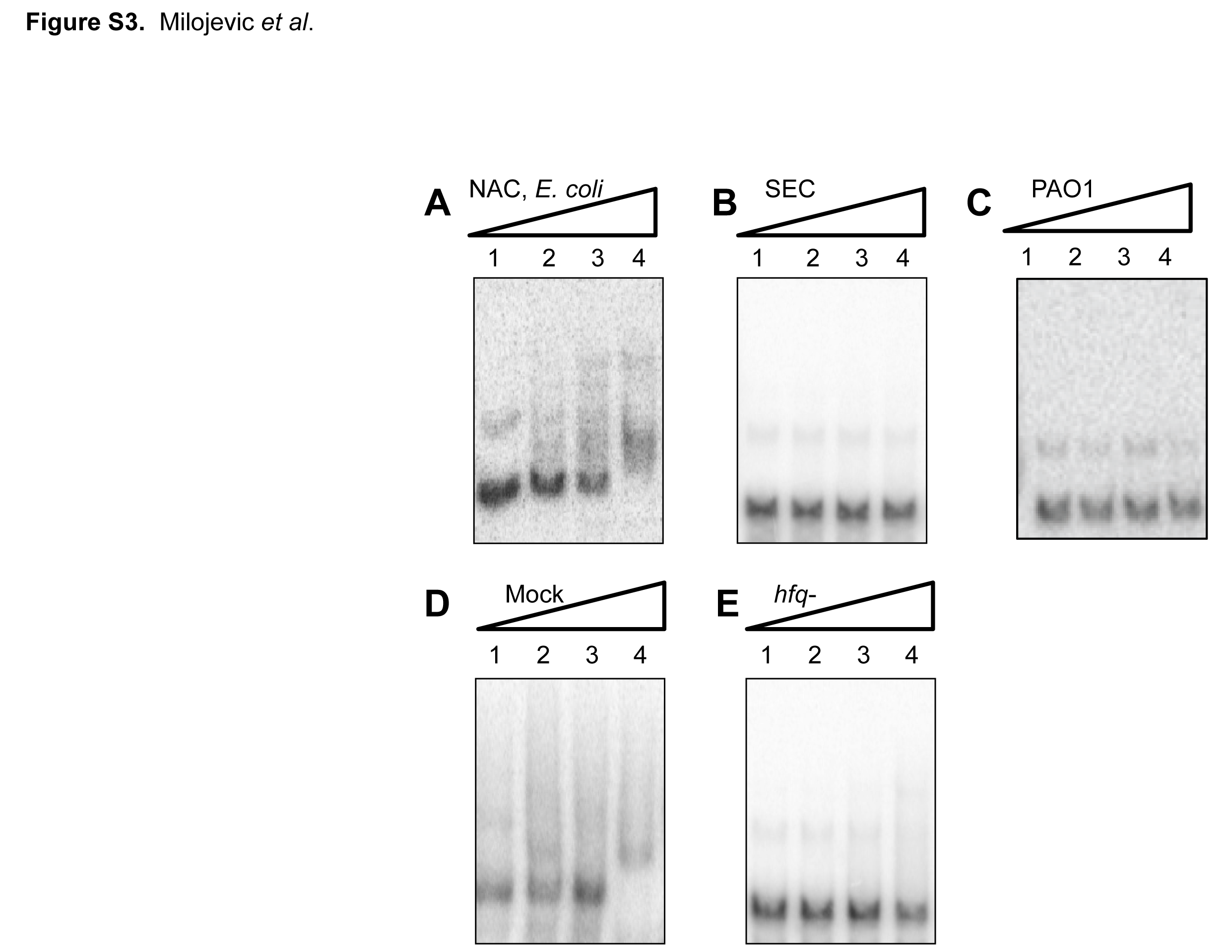

Supplement: Figure S3 — Electrophoretic mobility shift assay using amiÈ RNA. Electrophoretic mobility shift assay of 10 nM 5′-end labeled amiE′ RNA with increasing amounts of His-Crc purified from the E. coli strain Rosetta™ (DE3)(pLysS, pETM14lic-His6Crc) by one-step NAC (A) and by one-step NAC followed by SEC (B), respectively. EMSA assay employing the His-Crc protein purified from the P. aeruginosa strain PAO1(pME9670) by one-step NAC (C), the protein eluate obtained after one-step NAC from Rosetta™ (DE3)(pLysS, pETM14lic) (mock control; no Crc protein) (D) and the His-Crc protein from E. coli hfq- strain JW4130(pME9670) by one-step NAC (E). Lane 1, no protein was added to labeled amiE ` RNA. Lanes 2–4, the protein fractions were added in 50, 100 and 200-fold molar excess over labeled RNA. In the case of the mock preparation (D), the same amount of protein was added to RNA as in the experiments shown in panels A, B and C. (TIF) [file pone.0064609.s003.tif]

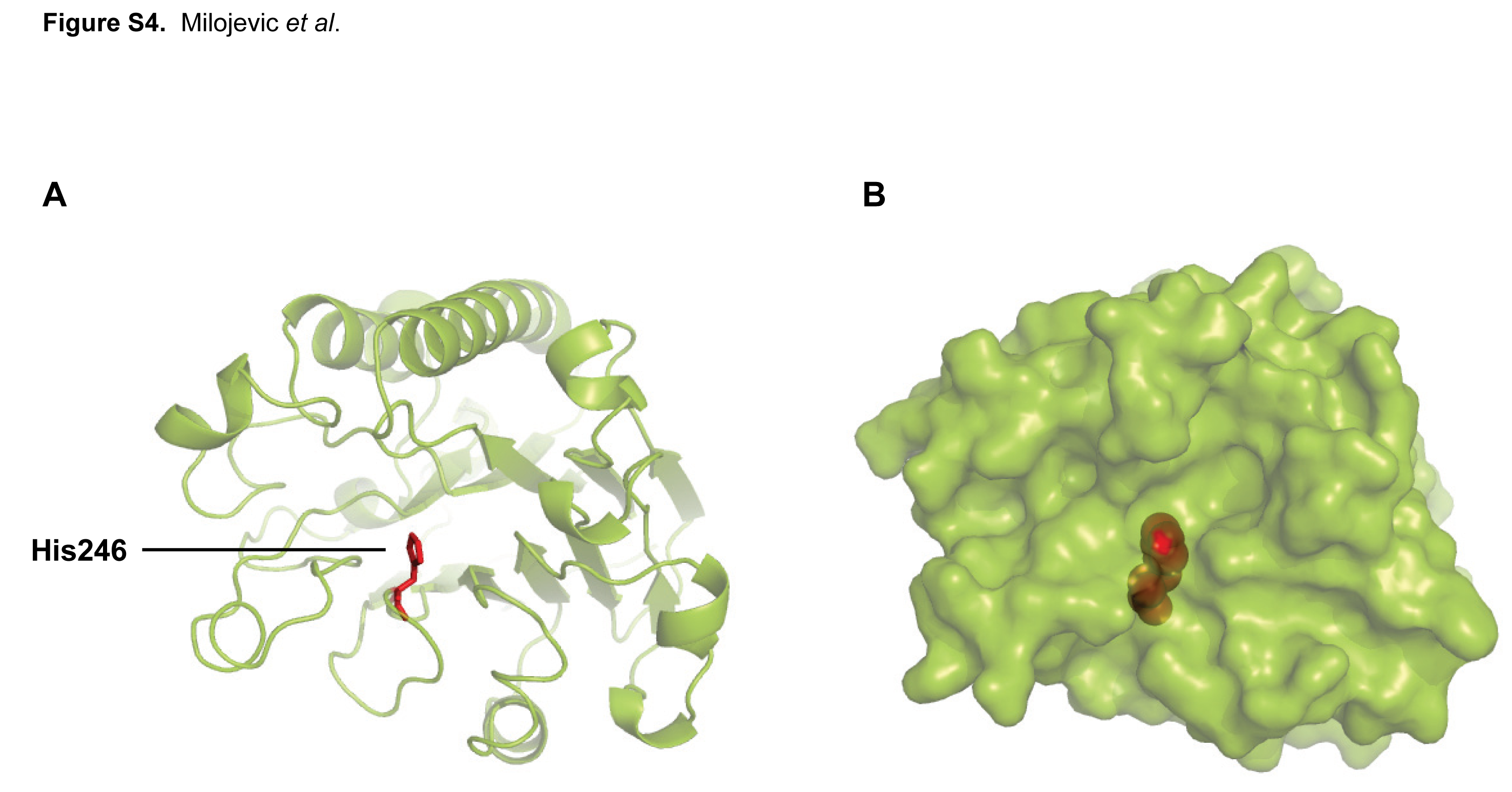

Supplement: Figure S4 — Ribbon diagram and surface of Crc highlighting the position of His246. The position of His246 is depicted in the ribbon diagram (A) and in the surface representation (B) of Crc, suggesting its side chain localization within a solvent exposed area. (TIF) [file pone.0064609.s004.tif]
